# Retinal OCT findings in acute central retinal artery occlusion of varying severity at different disease stages – a retrospective, observational study

**DOI:** 10.1186/s40942-023-00475-8

**Published:** 2023-06-22

**Authors:** Rubble Mangla, Ramesh Venkatesh, Rohini Sangoram, Isha Acharya, Yash Parmar, Vishma Prabhu, Naresh Kumar Yadav, Jay Chhablani

**Affiliations:** 1 Dept. of Retina and Vitreous, Narayana Nethralaya, #121/C, 1st R Block, Chord Road, Rajaji Nagar, 560010 Bengaluru, Karnataka India; 2 Medical Retina and Vitreoretinal Surgery, University of Pittsburgh School of Medicine, 203 Lothrop Street, Suite 800, Pittsburg, PA 15213 USA

**Keywords:** Central retinal artery occlusion, Temporal changes, Optical coherence tomography, Inner retina

## Abstract

**Purpose:**

To study the optical coherence tomography (OCT) changes in eyes with acute central retinal artery occlusion (CRAO) of different severity and at different disease stages.

**Methods:**

The study included acute CRAO cases of < 7 days duration, imaged on OCT at various time points. Based on the OCT findings at presentation, cases were classified into three severity groups: mild, moderate, and severe. OCT scans were evaluated and classified into four-time intervals based on symptom duration.

**Results:**

There were 39 eyes from 38 patients with acute CRAO who underwent 96 OCT scans. At presentation, the study had 11, 16, and 12 cases of mild, moderate, and severe CRAO, respectively. Middle retinal layer opacification was more common in mild CRAO cases, which caused inner retinal layer thinning over time. Moderate CRAO cases had total inner retinal layer opacification, which resulted in retinal thinning over time. Prominent middle limiting membrane (p-MLM) sign was seen in mild and moderate CRAO eyes while were not visualised in severe CRAO. This sign gradually faded out over time. Other OCT findings in higher grades of CRAO included inner retinal fluid, neurosensory detachment, internal limiting membrane detachment, hyperreflective foci, and posterior vitreous opacities. Regardless of the CRAO grade, the final end-point seen was inner retinal layer thinning over time.

**Conclusion:**

OCT in CRAO is a useful for determining the severity of retinal ischemia, disease stage, tissue damage mechanism, and final visual outcome. More prospective studies analysing a larger number of cases at fixed time points will be required in the future.

**Trial Registration:**

Trial Registration Number: Not applicable.

## Introduction

Albrecht von Graefes first described central retinal artery occlusion (CRAO) in 1859 as a disastrous ophthalmic emergency characterized by sudden onset, acute painless loss of vision, and a poor prognosis [1]. All cases of acute CRAO on fundus examination reveal extensive retinal whitening, presence of a cherry red spot, retinal vessel attenuation, and preservation of optic nerve head perfusion. In some cases, however, the exam reveals a patent cilioretinal artery perfusion and/or a visible embolus within the central retinal artery [2]. The presenting visual acuity in these patients is influenced by a number of variables. These include the degree of acute retinal ischemia, changes detected on optical coherence tomography (OCT) in the inner layers of the retina, and the presence of a cilioretinal artery and its perfusion to the papillomacular bundle [3–7].

According to Hayreh, CRAO mainly affects the inner retinal layers [8]. OCT has been an invaluable, non-invasive imaging tool in identifying a variety of inner and outer retinal structural features in CRAO [5, 6, 9, 10]. In routine clinical practice, OCT findings such as presence of inner retinal layer hyperreflectivity, thickening, and loss of inner retinal layer stratification are the common findings used for the diagnosis of acute CRAO on OCT [6]. While the chronic stage of the disease is identified by the presence of a thinner inner retina and preservation of inner retinal layer stratification [11]. The OCT findings in CRAO can sometimes cause diagnostic dilemmas as well. The most frequently encountered ones are the presence of the predominant middle retinal layer opacification, which can be seen in cases of resolving CRAO, paracentral acute middle maculopathy, or occasionally in cilioretinal artery occlusion. Similar dilemma is seen in eyes with inner retinal layer thinning, which can occur after chronic glaucoma or after CRAO resolution [12]. OCT changes following an acute CRAO at different time points may explain temporal changes. To the best of our knowledge, there is not enough information in the literature at present discussing the changes on OCT in acute CRAO eyes at different stages of the disease.

This study essentially detects and describes the various OCT features in eyes with acute CRAO who have had symptoms for < 7 days duration and studies OCT changes over the course of 30 days.

## Methods

Patients diagnosed with acute CRAO with duration of onset of symptoms < 7 days and examined on OCT were included for this retrospective study. An acute obstruction of central retinal artery was defined as recent history of sudden, painless loss of vision with presence of retinal thickening and whitening, retinal vessel attenuation and a cherry red spot at the macula. The study included patients who visited the retina clinic between June 2017 and November 2022. The study complied with the tenets of the Declaration of Helsinki and was approved by the local Institutional Review Board/Ethics Committee. Because the study was a retrospective analysis, waiver for informed consent was obtained.

**Fig. 1:**
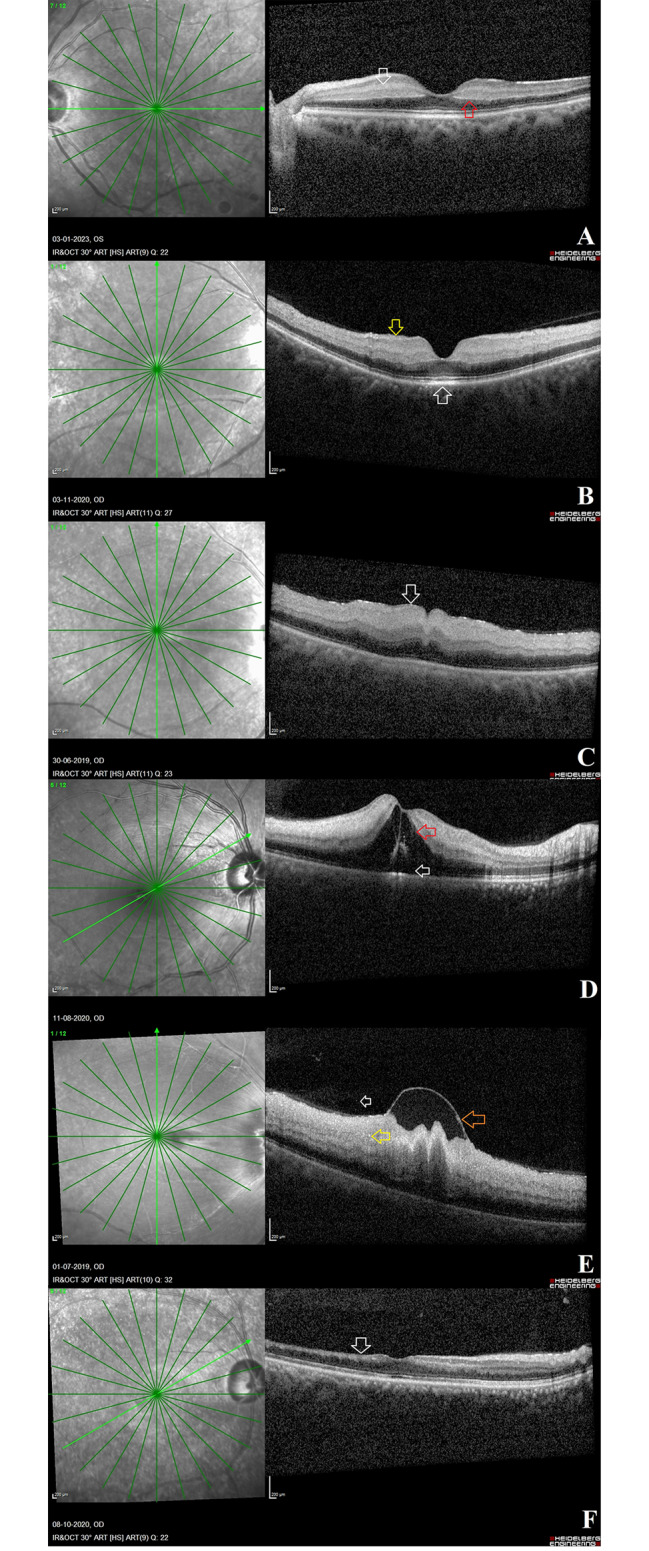
**OCT findings seen in eyes with CRAO at different stages of the disease**: **Fig. 1A**: Middle retinal layer opacification (white arrow) and prominent middle limiting membrane (p-MLM) sign (red arrow). **Fig. 1B**: Inner retinal layer opacification (yellow arrow) and prominent retinal pigment epithelium at the fovea (white arrow). **Fig. 1C**: Inner retinal layer opacification (white arrow) with inner retinal layer thickening with absent inner retinal layer stratification. **Fig. 1D**: Inner retinal layer hypo reflective fluid-filled cavities (red arrow) and neurosensory detachment (white arrow). **Fig. 1E**: Internal limiting membrane detachment (orange arrow) with hyperreflective posterior vitreous opacities (white arrow) and inner retinal hyperreflective foci (yellow arrow). **Fig. 1F**: Inner retinal layer thinning (white arrow)

Medical records for all these acute CRAO cases were reviewed, and demographic and ophthalmic data were compiled. Age, gender, time interval between onset of symptoms and presentation to clinic, presence of associated systemic disease if any, and Snellen’s visual acuity in the affected eye at every visit were all documented. The presence of a patent cilioretinal artery was determined using colour fundus photographs. The Spectralis HRA OCT system (Heidelberg Engineering, Heidelberg, Germany) was used to obtain a high-resolution spectral-domain OCT scan with 25-line horizontal volume scans covering the area centred on the fovea. The descriptive characteristics that were noted on OCT from inside to outside included the presence of posterior vitreous opacities, inner retinal layer features such as inner limiting membrane detachment (ILMD), hyperreflectivity, thickening and loss of individual layer stratification, hyperreflective foci, hyporeflective fluid-filled cavities, presence of prominent middle limiting membrane (p-MLM) sign, presence of neurosensory detachment, and prominent retinal pigment epithelium (RPE) at the fovea (Fig. 1). On the basis of the OCT findings, the severity of CRAO was categorised into mild, moderate and severe occlusion. Descriptions of the mild, moderate and severe grades of CRAO is mentioned in Table 1.

**Table 1:** Severity of acute CRAO based on OCT features:

	Middle +/- Inner retinal layer opacification	Inner retinal layer thickening	Absent inner retinal layer stratification
Mild	Yes	No	No
Moderate	Yes	Yes	No
Severe	Yes	Yes	Yes

Abbreviations: CRAO – central retinal artery occlusion; OCT – optical coherence tomography

Patients with a history of ocular trauma, macular disease, vitreomacular interface disorders, severe non-proliferative or proliferative diabetic retinopathy, ocular surgery other than cataract surgery, or high myopia were not included in the study. For these study patients, follow-up OCT scans were evaluated for the same set of features. For easier analysis and understanding, OCT scans were classified into 4 categories based on the time interval between the symptom onset (Day 0) and OCT scan visit date. The four categories were ≤ 7, 8–14, 15–30 and > 30 days respectively.

### Statistical analysis

All data were analysed using GraphPad Prism version 9.5.0 (730) for Windows, GraphPad Software, San Diego, California USA, www.graphpad.com. The vision data at presentation was documented as Snellen’s vision data for the study. The visual acuity for the three different grades of acute CRAO was noted as a range in the study. The proportion of different OCT imaging features were described as numbers and percentages. Chi-square test was used to compare the categorical data between 2 or more groups. P values < 0.05 were considered statistically significant.

## Results

This observational, retrospective, cross-sectional study at different time points included 39 eyes of 38 patients diagnosed with acute CRAO of varying severity and duration of symptoms ≤ 7 days. There were 32 (84%) males and 6 (16%) females included in the study analysis. The median age of the study patients was 49.5 years with an interquartile range from 35.0 to 63.5 years. 61% (n = 23) of the CRAO patients had at least one associated systemic condition such as diabetes mellitus, cardiac illness, hypertension, stroke or hyperhomocysteinemia. Right eye was affected in 25 (64%) cases and left eye in 14 (36%) cases respectively. Visual acuity at presentation ranged from light perception (PL + ve) to 6/6 based on the severity of retinal artery occlusion. The median time interval from symptom onset to presentation to clinic was 1 day with an interquartile range from 1 to 4 days. Table 2 shows the demographic and clinical details of acute CRAO cases included in the study. A patent cilioretinal artery was identified in 11 (28%) eyes.

**Table 2:** Demographic findings of patients with different grades of severity of CRAO:

	Mild CRAO	Moderate CRAO	Severe CRAO
No. of eyes (n, %)	11 (28)	16 (41)	12 (31)
Laterality (RE: LE)	6:5	14:2	5:7
Age [years] (mean ± SD)	46.73 ± 20.36	49.56 ± 13.03	50.83 ± 19.73
Gender (Males) [n, %]	9 (82)	12 (75)	12 (100)
Associated systemic illness (n, %)	5 (45)	10 (63)	8 (75)
Snellen visual acuity at presentation (range)	FC @ 1 m – 6/6	PL + ve – 6/120	PL + ve – 6/60
Interval from symptom onset to presentation to clinic (days)	2.909 ± 2.548	2.125 ± 1.928	3.0 ± 2.174
Presence of patent CLRA (n, %)	2 (18)	4 (25)	5 (42)

Abbreviations: CRAO – central retinal artery occlusion; RE – right eye; LE – left eye; CLRA – cilioretinal artery

In all, 96 OCT visit scans were available for these 39 acute CRAO eyes at different stages of the disease. The number of OCT scans available for analysis in the mild, moderate and severe CRAO grades were 23, 44 and 29 respectively at different time points.

The OCT findings seen at different time points for varying severity of acute CRAO is mentioned in Table 3.

**Table 3:** OCT findings at different time points with varying grades of acute CRAO:

Severity of CRAO	Mild CRAO (n = 11)				Moderate CRAO (n = 16)				Severe CRAO (n = 12)			
Days from the onset of symptoms	< 7 (n = 13)	8–14 (n = 4)	15–30 (n = 1)	> 30 (n = 5)	< 7 (n = 26)	8–14 (n = 7)	15–30 (n = 4)	> 30 (n = 7)	< 7 (n = 20)	8–14 (n = 3)	15–30 (n = 2)	> 30 (n = 4)
Total inner retinal layer opacification (n, %)	3 (23)	1 (25)	0 (0)	0 (0)	23 (89)	4 (57)	2 (50)	2 (29)	20 (100)	2 (66)	1 (50)	2 (50)
P value	0.633				0.01				0.013			
Middle retinal layer opacification alone (n, %)	10 (77)	2 (50)	0 (0)	0 (0)	3 (11)	3 (43)	2 (50)	2 (29)	0 (0)	0 (0)	0 (0)	0 (0)
P value	0.021				0.151				-			
Loss of inner retinal layer stratification (n, %)	0 (0)	0 (0)	0 (0)	0 (0)	3 (11)	1 (14)	0 (0)	0 (0)	11 (55)	3 (100)	2 (100)	2 (50)
P value	-				0.678				0.292			
Inner retinal layer fluid filled cavities (n, %)	0 (0)	0 (0)	0 (0)	0 (0)	7 (27)	3 (43)	1 (25)	1 (14)	5 (25)	1 (33)	0 (0)	1 (25)
P value	-				0.691				0.853			
Neurosensory detachment (n, %)	0 (0)	0 (0)	0 (0)	0 (0)	6 (23)	2 (29)	0 (0)	1 (14)	1 (5)	0 (0)	0 (0)	0 (0)
P value	-				0.663				0.926			
Internal limiting membrane detachment (n, %)	0 (0)	0 (0)	0 (0)	0 (0)	5 (19)	3 (43)	0 (0)	1 (14)	5 (25)	1 (33)	0 (0)	1 (25)
P value	-				0.337				0.853			
Posterior vitreous cells (n, %)	0 (0)	0 (0)	0 (0)	0 (0)	7 (27)	1 (14)	1 (25)	0 (0)	4 (20)	0 (0)	0 (0)	1 (25)
P value	-				0.443				0.725			
Hyperreflective RPE at the fovea (n, %)	6 (46)	1 (25)	0 (0)	0 (0)	23 (89)	5 (71)	1 (25)	1 (14)	15 (75)	1 (33)	0 (0)	0 (0)
P value	0.241				< 0.001				0.011			
Prominent middle limiting membrane (n, %)	10 (77)	4 (100)	1 (100)	2 (40)	19 (73)	3 (43)	1 (25)	2 (29)	1 (5)	0 (0)	0 (0)	0 (0)
P value	0.186				0.019				0.926			
Hyperreflective foci (n, %)	0 (0)	0 (0)	0 (0)	0 (0)	0 (0)	0 (0)	1 (25)	2 (29)	2 (10)	0 (0)	0 (0)	2 (50)
P value	-				0.021				0.142			
Inner retinal layer thinning (n, %)	0 (0)	1 (25)	1 (100)	5 (100)	0 (0)	0 (0)	0 (0)	3 (43)	0 (0)	1 (33)	1 (50)	2 (50)
P value	< 0.001				< 0.001				0.013			

Abbreviations: CRAO – central retinal artery occlusion; RPE – retinal pigment epithelium

### OCT changes in patients with mild CRAO at different time points (Fig. 2)

**Fig. 2:**
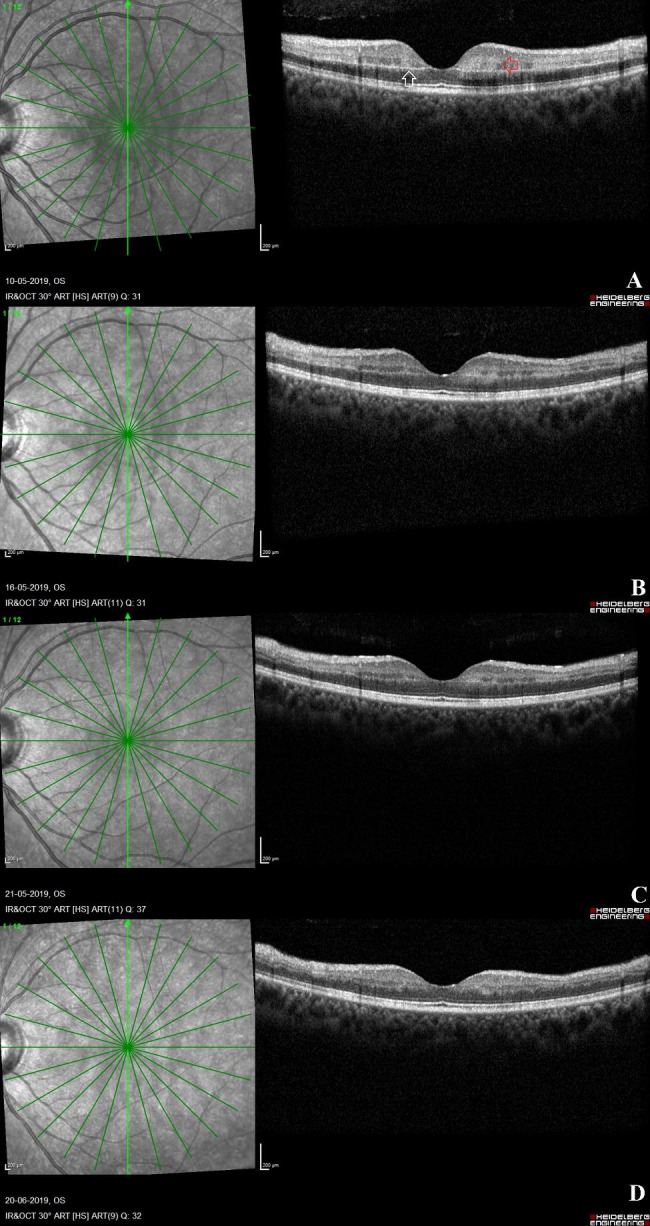
**OCT scans in a case of acute mild CRAO**: **Fig. 2A**: The left eye OCT scan in a case of acute CRAO at four-day interval from the onset of visual symptoms shows the opacification of the middle retinal layers (red arrow) along the length of the scan with a hyperreflective line seen at the inner boundary of the outer plexiform layer to suggest the presence of a prominent middle limiting membrane (p-MLM) sign (white arrow). There is no thickening of the inner retinal layers and the individual inner retinal layers can easily be delineated. These OCT features are suggestive of a mild variety of acute CRAO. **Fig. 2B**: On day 10, post presentation of symptoms, the OCT scan shows reduction in middle retinal layer opacification. **Fig. 2C** and **D**: On days 15 and 45 post presentation of symptoms, there is thinning of the middle retinal layers and intact stratification of the inner retinal layers

In this study, 11 acute CRAO eyes with mild severity were identified. During the first week following the onset of symptoms, the most prominent finding on the OCT scan was opacification of the middle retinal layers (n = 10, 77%) throughout the scan length. Furthermore, at the acute stage of the disease, 3 (23%) eyes had patchy opacification of the innermost retinal layers. At presentation, the retina at the posterior pole was not thickened, and individual inner retinal layer differentiation was possible in all eyes. Within the first week of symptoms, a p-MLM sign was seen in 10 of the 13 (77%) eyes with mild CRAO. The presence of a prominent hyperreflective RPE layer at the fovea (n = 6, 46%) was less common in eyes with mild CRAO compared to the more severe grades of the disease. By the second week following the onset of symptoms, there was normalisation of middle and innermost retinal layer opacification and thinning of the inner retinal layers. The p-MLM sign was still visible during this time period. Further thinning of the inner retinal layers was observed in the only OCT scan available between the 15-30-day interval. This eye revealed the presence of a p-MLM sign. After 1 month of visual symptom onset, there was thinning of the inner retinal layers and persistence of p-MLM sign on OCT. Other OCT findings, such as inner retinal fluid-filled hyporeflective cavities, neurosensory detachment, internal limiting membrane detachment, inner retinal hyperreflective foci and posterior vitreous hyperreflective opacities were not seen in any of the eyes with mild acute CRAO across the study.

### OCT changes in patients with moderate CRAO at different time points (Fig. 3)

**Fig. 3:**
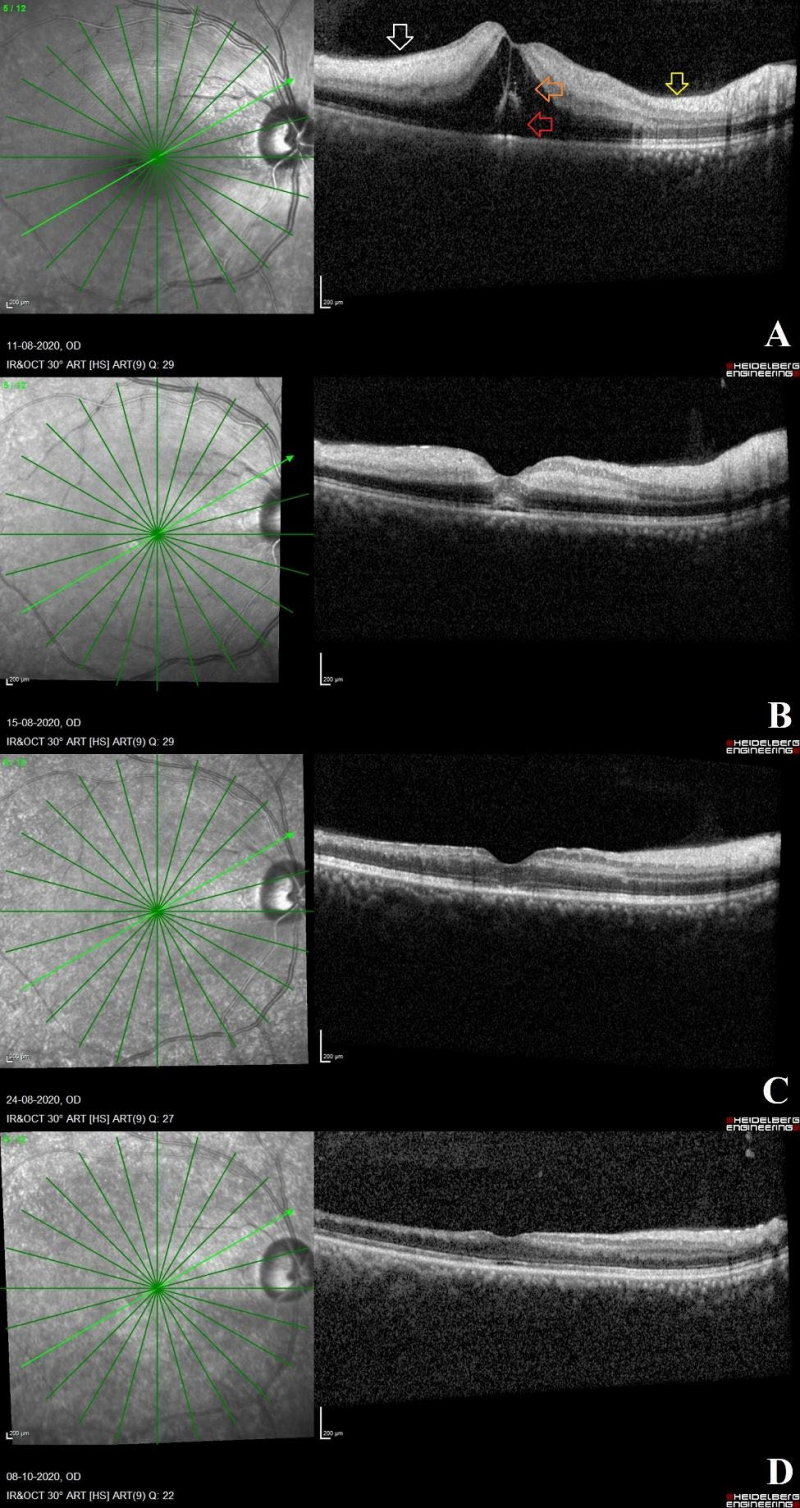
**OCT scans in a case of acute moderate CRAO**: **Fig. 3A**: The right eye OCT scan in a case of acute CRAO at five-day interval from the onset of visual symptoms shows the opacification and thickening of the inner retinal layers (white arrow) with the presence of inner retinal hyporeflective fluid filled cavities (orange arrow) and small pocket of neurosensory detachment (red arrow) and prominent hyperreflectivity at the fovea. The retinal layer stratification is still intact. These OCT features are suggestive of a moderate variety of acute CRAO. In addition, a portion of the OCT scan appears normal on the temporal aspect of the optic disc on the papillomacular bundle suggesting of a patent cilioretinal artery (yellow arrow). **Fig. 3B**: On day 9, post presentation of symptoms, the OCT scan shows reduction in inner retinal layer opacification with middle retinal layer opacification being prominent. The inner retinal edema and neurosensory detachment have resolved and a normal foveal contour has been achieved. **Fig. 3C** and **D**: On days 18 and 58 post presentation of symptoms, there is extensive thinning of the inner retinal layers with intact stratification of the individual retinal layers

In this study, 16 acute CRAO eyes with moderate severity were included. In the first week after the onset of visual symptoms in these eyes, the most common OCT retinal findings were inner retinal opacification and increased retinal thickness. Retinal layer stratification was still possible in most eyes of moderate CRAO during this period. Other common OCT findings during this disease stage included a prominent hyperreflective RPE at the fovea and a p-MLM sign. In the acute stages of moderate CRAO eyes, other less common findings included altered foveal contour, inner retinal fluid-filled hyporeflective cavities, neurosensory detachment, internal limiting membrane detachment, and posterior vitreous hyperreflective opacities. During the second week, since the onset of symptoms, total inner retinal layer opacification had decreased and the retinal thickness had reduced significantly, resulting in a flatter foveal contour. Over time, i.e., on OCT scans > 30-day duration of symptoms, there was thinning of the inner retinal layers and flat, altered foveal contour in chronic stages of the disease. Other OCT findings such as inner retinal fluid-filled hyporeflective cavities, neurosensory detachment, internal limiting membrane detachment, inner retinal hyperreflective foci and posterior vitreous hyperreflective opacities were also seen in eyes with moderate CRAO.

## OCT changes in patients with severe CRAO at different time points (Fig. 4)

**Fig. 4:**
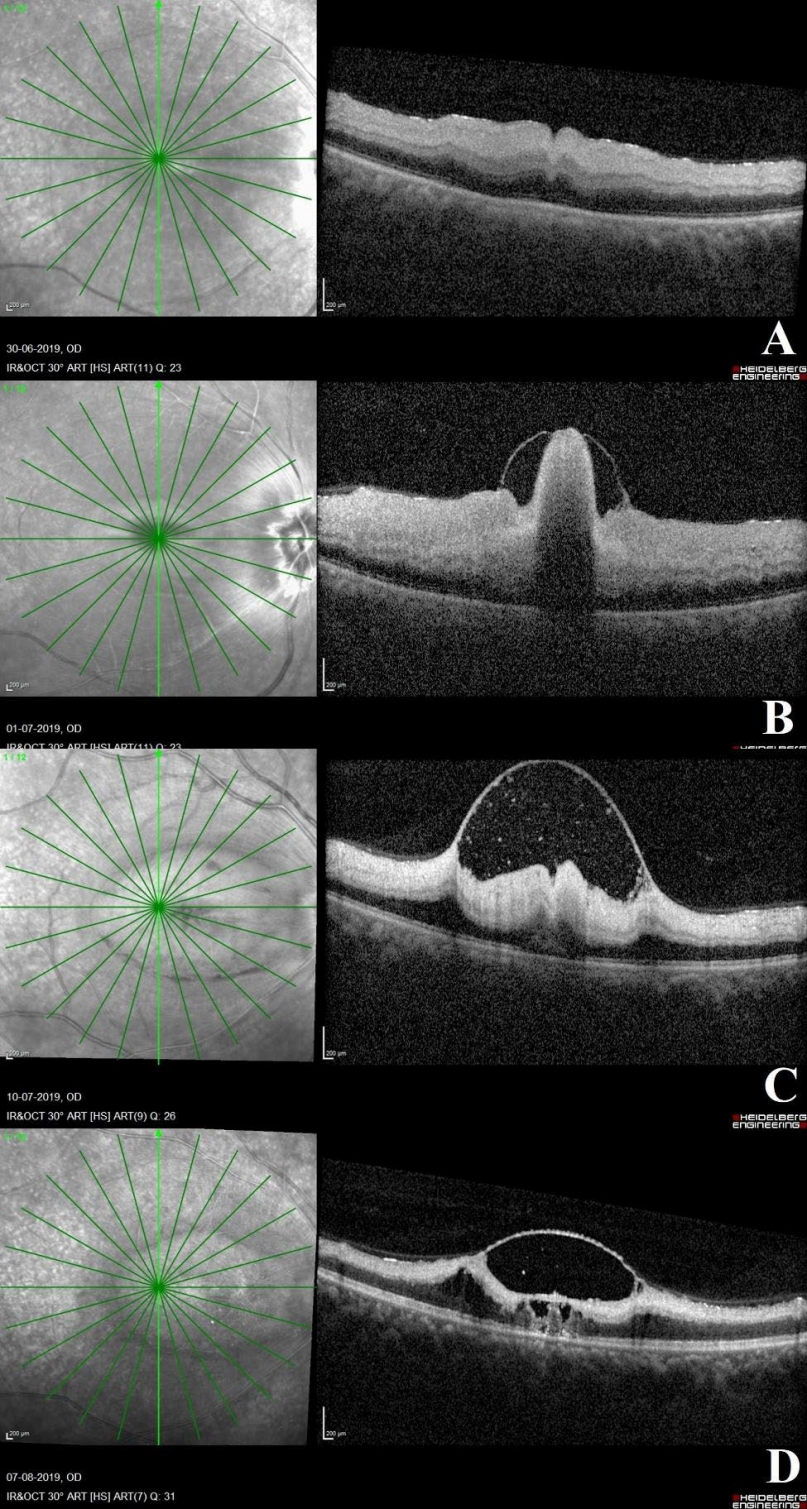
**OCT scans in a case of acute severe CRAO**: **Fig. 4A**: The right eye OCT scan in a case of acute CRAO at 2-day interval from the onset of visual symptoms shows the opacification and thickening of the inner retinal layers and loss of individual inner retinal layer stratification. These OCT features are suggestive of a severe variety of acute CRAO. **Fig. 4B**: OCT scan done on the following day shows the separation of the internal limiting membrane (ILM) from the underlying nerve fibre layer with multiple hyperreflective posterior dots suggestive of internal limiting membrane detachment. There is still inner retinal layer opacification, thickening and loss of retinal layer stratification. The normal foveal contour is lost. **Fig. 4C**: On day 13, post presentation of symptoms, the OCT scan shows reduction in inner retinal layer opacification and thickening. There is persistence of the ILM detachment with multiple sub-ILM hyperreflective clumps. with middle retinal layer opacification being prominent. The inner retinal edema and neurosensory detachment have resolved and a normal foveal contour has been achieved. **Fig. 4D**: On day 41 post presentation of symptoms, there is extensive thinning of the inner retinal layers, with ILM separation and sub-ILM hyperreflective spots, necrosis of the retinal layers and tenting of the retinal layers due to ILM detachment

Twelve eyes with acute severe CRAO eyes were included. During the acute stage of the disease, within one week of the appearance of visual symptoms, the most common findings were total inner retinal opacification in all cases with significant retinal thickening, loss of inner retinal layer differentiation, and prominent RPE hyperreflectivity at the fovea. Over the course of the follow-up, there was a reduction in retinal thickness. Other OCT findings in some eyes with severe acute CRAO included inner retinal fluid-filled hyporeflective cavities, neurosensory detachment, internal limiting membrane detachment, inner retinal hyperreflective foci, posterior vitreous hyperreflective opacities, and focal photoreceptor layer defects at the fovea. When compared to less severe grades of CRAO, these findings were more common in the acute stages of severe CRAO eyes.

**Fig. 5:**
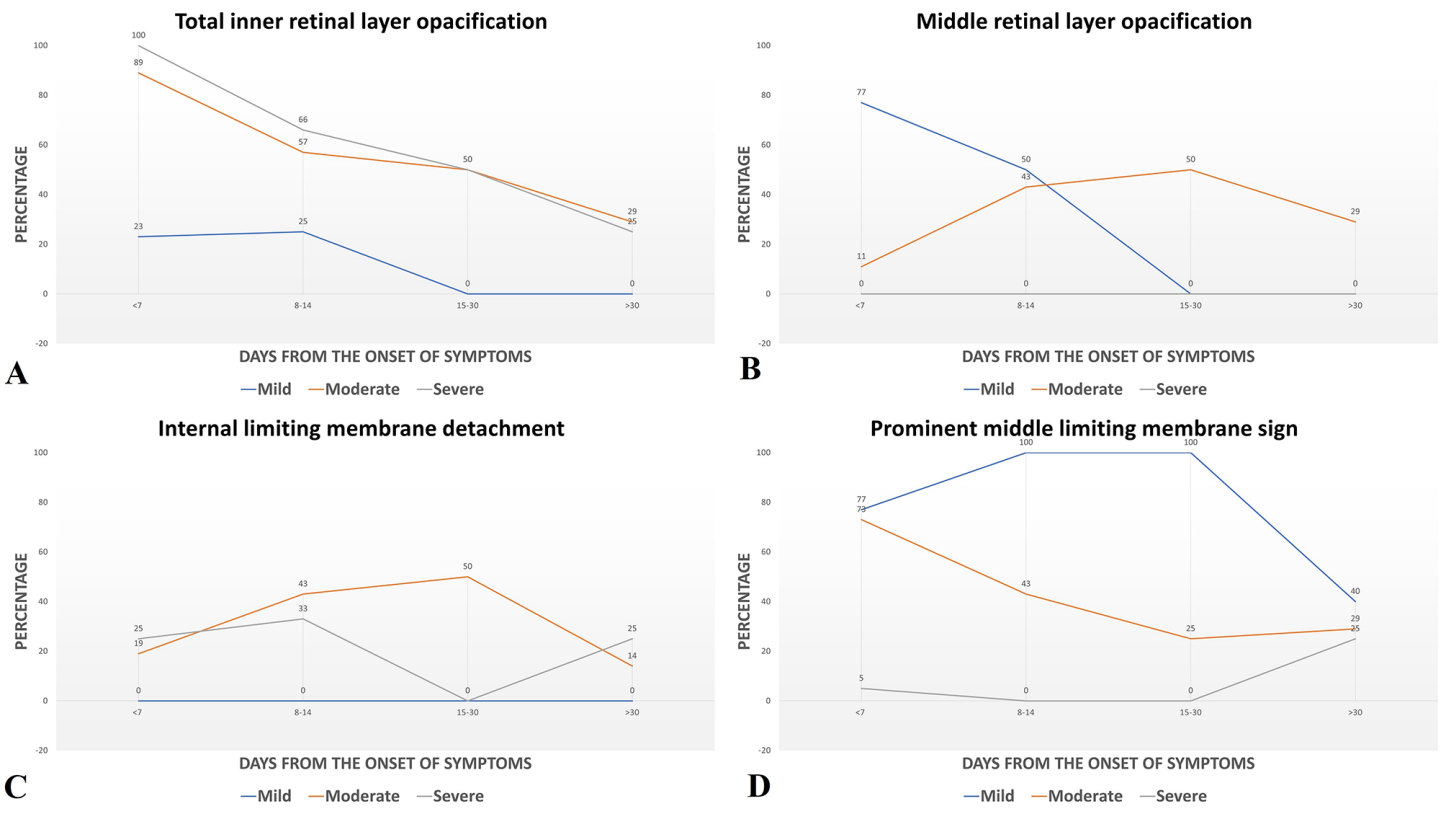
**Graph depicting the various OCT signs for different grades of acute CRAO**: **Fig. 5A**: Graph depicting the inner retinal layer opacification changes on OCT at different time points in eyes with varying grades of acute CRAO. **Fig. 5B**: Graph depicting the middle retinal layer opacification changes on OCT at different time points in eyes with varying grades of acute CRAO. **Fig. 5C**: Graph depicting the internal limiting membrane detachment changes on OCT at different time points in eyes with varying grades of acute CRAO. **Fig. 5D**: Graph depicting the prominent middle limiting membrane sign changes on OCT at different time points in eyes with varying grades of acute CRAO

Figure 5 depicts the OCT changes of different important signs for varying grades of CRAO at different time points.

## Discussion

This study concentrated on the various OCT findings seen in eyes with varying degrees of acute CRAO at various time points and disease stages. According to our findings, the initial OCT finding in mild cases of acute CRAO was predominant opacification of the middle retinal layers. Higher grades of CRAO had complete opacification of the inner retinal layers, as well as increased retinal thickening and a lack of stratification of the individual retinal inner layers, as seen on OCT. Regardless of the severity grade of acute CRAO, inner retinal thinning was the common endpoint over time. The p-MLM sign was well-visible in acute stages of mild and moderate CRAO, but faded as symptoms lasted longer. In severe cases of acute CRAO, the p-MLM sign was not seen. The p-MLM sign in severe CRAO did not become visible over time. In mild acute CRAO cases, OCT findings such as inner retinal fluid-filled hyporeflective cavities, neurosensory detachment, ILMD, inner retinal hyperreflective foci, and posterior vitreous hyperreflective opacities were absent. These findings were more noticeable in moderate to severe grades of acute CRAO, and in the severe chronic form of the disease, these changes lasted longer even after the retinal opacification and inner retinal thinning resolved.

The middle retinal layer opacification and p-MLM sign, we believe, are the first changes to appear in eyes with mild acute cases of CRAO. The neurosensory retina’s anatomy suggests the presence of two major microvascular capillary plexuses (superficial and deep), three major cell bodies, and two synaptic plexuses. Anatomically, the neurosensory retina is divided into three broad regions: outer retina (comprising of rod and cone layers, external limiting membrane, and outer nuclear layer), middle retina (comprising of outer plexiform layer, inner nuclear layer, and inner plexiform layer), and inner retina (comprising of ganglion cell layer, nerve fibre layer and internal limiting membrane) [13, 14]. The middle retinal layer is made up primarily of two synaptic plexuses, a dense deep microvascular capillary plexus, and the cell bodies of bipolar cells, horizontal cells, and amacrine cells [15]. In comparison to the superficial capillary plexus which is located in the retinal nerve fibre layer, the deep capillary plexus is a denser capillary network and is predominantly supplied by the central retinal artery via the superficial vascular plexus [15]. When the central retinal artery suddenly gets hypo perfused, the perfusion of the deep capillary plexus gets primarily affected along with the superficial capillary plexus, resulting in cellular swelling and extracellular edema predominantly in the middle retinal layers, which causes its opacification [2]. As a result, with a milder grade of CRAO, the opacification is limited to the middle retinal layers, with no significant retinal thickening or loss of retinal layer stratification. While the severity of the occlusion increases, the superficial capillary plexus becomes involved as well, resulting in opacification of the inner retinal layers, retinal thickening, and the absence of inner retinal layer opacification [16]. Cell body death occurs over time, resulting in retinal layer thinning and preservation of retinal layer stratification. The retinal opacification fades out depending on the severity of the occlusion, with the opacification in the middle retinal layers being the last to resolve [16]. Furashova et al. classified acute CRAO into incomplete, subtotal, and total varieties based on the presence of inner retinal opacification with/without inner retinal layer thickening or absence of inner retinal layer stratification [5]. According to the current study, middle retinal layer opacification and p-MLM signs appear on OCT even earlier in a case of acute CRAO. Furashova et al. did not consider middle retinal layer opacification or p-MLM signs in their study [5]. As a result, we believe that instead of the previously described nomenclature of incomplete, subtotal, and total CRAO, the OCT classification of acute CRAO should be adjusted to mild, moderate, and severe grades.

The bipolar cell synapses are located in the MLM, which is located at the inner boundary of the outer plexiform layer [17]. In mild and moderate grades of acute CRAO, there is acute swelling predominantly of bipolar cell synapses, resulting in a hyperreflective line on OCT suggestive of a p-MLM sign. All cells of the inner retinal layers suffer from acute swelling in severe acute CRAO, resulting in hyperreflectivity of the entire inner retinal layers on OCT. As a result of the higher reflectivity of the other inner retinal layers, the p-MLM sign is no longer visible on OCT. As a result, in acute CRAO, the p-MLM sign is an indicator of less severe ischemic damage and may be a factor associated with a better visual acuity outcome. Our observations on the p-MLM sign are similar to that reported by Chu et al. and Schneider et al. [18, 19].

Other signs seen on OCT in this study included inner retinal fluid-filled hyporeflective cavities, neurosensory detachment, ILMD, inner retinal hyperreflective foci, and posterior vitreous hyperreflective opacities, in addition to inner retinal opacification, thickening, and loss of individual retinal layer stratification. Based on the concept of reperfusion injury seen with acute CRAO, these signs can be explained as follows: Once the critical duration of ischemia is exceeded in acute CRAO, retinal cell injury and/or death occur. The magnitude and duration of ischemia influence the extent of cell dysfunction, injury, and/or death. Ischemia can cause cell swelling and rupture, as well as cell death via necrotic, necroptotic, apoptotic, and autophagic mechanisms [20]. Although prompt reperfusion restores oxygen and substrate delivery, reperfusion appears to have negative consequences due to an increase in reactive oxygen species generation and proinflammatory neutrophilic infiltration of the ischemic tissues. This is known as reperfusion injury [21, 22]. As a result of severe retinal ischemia and reperfusion injury, retinal cell injury and/or death can occur following acute CRAO. The tissue injury caused by retinal ischemia and reperfusion increases proportionally to the magnitude of ischemia caused by CRAO. The retinal tissue undergoes necrosis and apoptosis, resulting in intraretinal degeneration, permanent necrosis, and atrophy. As a result, the ILM is unsupported, resulting in an ILMD [9, 23]. There is an increase in reactive oxygen radicals and proinflammatory macrophage like cells in the retinal tissue during reperfusion injury, which can be identified as hyperreflective dots in the inner retinal layers, sub-ILM space, or posterior cortical vitreous [9, 24, 25]. Increased inflammation following retinal reperfusion may result in the breakdown of the inner blood retinal barrier, causing fluid accumulation in the retinal layers as cystoid macular edema or in the subretinal space as neurosensory detachment [26, 27]. These OCT findings are usually indicative of more severe retinal ischemia and are considered indicators of poor visual outcomes. These changes are associated with significant inner retinal thinning and destruction over time, explaining why such eyes have poor visual outcomes.

On OCT, the middle retinal layer opacification seen in less severe forms of acute CRAO is frequently confused with the retinal opacification seen in paracentral acute middle maculopathy or cilioretinal artery occlusion. The retinal opacification in paracentral middle maculopathy is limited only to the middle retinal layers and can only be seen in the paracentral macular region [28]. The retinal opacification in cilioretinal artery occlusion is usually seen on the temporal aspect of the optic disc, extending temporally for a variable distance towards the macula depending on the length of the cilioretinal artery [29]. Another point of contention could be between the inner retinal layer thinning seen in the chronic stages of CRAO and the advanced stages of chronic glaucoma. The retinal thinning in chronic glaucoma is uniform, primarily affecting the ganglion cell layer and retinal fibre layer in the inner retina [12]. The retinal thinning seen in the chronic stages of CRAO, on the other hand, affects the inner and middle retinal layers, causing significant retinal layer thinning depending on the magnitude of retinal ischemia.

There are some limitations to the study. The study’s major limitation is its retrospective design. Second, not all patients’ scans were studied at the same defined time points. Instead, the scans were categorised based on the time since the visual symptoms, which was measured in days rather than weeks. These limitations can be overcome by designing a well-organized large sample, prospective study of an equal number of CRAO cases of varying severity, with OCT scans acquired at fixed time points for all patients and correlating the results with final visual outcome and other variables. Despite these limitations, the study provides valuable information on OCT changes in eyes suffering from acute CRAO.

Finally, OCT in CRAO provides information about the severity of retinal ischemia, disease stage, tissue damage mechanism, and acts as a predictor for final visual outcome. In the future, more prospective studies analysing a larger number of OCT scans at fixed time points will be required.

## Data Availability

The datasets used and/or analysed during the current study are available from the corresponding author on reasonable request.

## Declarations

### Animal research

This article does not contain any studies with animals performed by any of the authors.

## Abbreviations

CRAO: Central retinal artery occlusion
OCT: Optical coherence tomography
P-MLM: Prominent middle limiting membrane
ILM: Internal limiting membrane
ILMD: Internal limiting membrane detachment
RPE: Retinal pigment epithelium
